# Single-Species Microarrays and Comparative Transcriptomics

**DOI:** 10.1371/journal.pone.0003279

**Published:** 2008-09-25

**Authors:** Frédéric J. J. Chain, Dora Ilieva, Ben J. Evans

**Affiliations:** 1 Center for Environmental Genomics, Department of Biology, McMaster University, Hamilton, Ontario, Canada; 2 Michael DeGroote School of Medicine, McMaster University, Hamilton, Ontario, Canada; Minnesota State University Mankato, United States of America

## Abstract

**Background:**

Prefabricated expression microarrays are currently available for only a few species but methods have been proposed to extend their application to comparisons between divergent genomes.

**Methodology/Principal Findings:**

Here we demonstrate that the hybridization intensity of genomic DNA is a poor basis on which to select unbiased probes on Affymetrix expression arrays for studies of comparative transcriptomics, and that doing so produces spurious results. We used the Affymetrix *Xenopus laevis* microarray to evaluate expression divergence between *X. laevis*, *X. borealis*, and their F1 hybrids. When data are analyzed with probes that interrogate only sequences with confirmed identity in both species, we recover results that differ substantially analyses that use genomic DNA hybridizations to select probes.

**Conclusions/Significance:**

Our findings have implications for the experimental design of comparative expression studies that use single-species microarrays, and for our understanding of divergent expression in hybrid clawed frogs. These findings also highlight important limitations of single-species microarrays for studies of comparative transcriptomics of polyploid species.

## Introduction

Microarrays designed for one species have been used to explore expression divergence between species [1]–[10]. These studies deploy different types of microarrays on species with varying levels of divergence, and these experimental variables influence the potential for technical bias. In particular, the designs of experiments that deploy two-color versus one-color microarrays differ, and therefore can be differently subject to technical bias when these arrays are used to comparative expression between species. Microarrays with short oligonucleotide probes might be more profoundly impacted by a single base pair mismatch than ones with longer oligonucleotides. Additionally, studies of species that are substantially diverged have more sequence differences and other possible sources of variation (alternative splicing, repetitive elements, duplications, etc.) that increase the chance of technical bias. Differences in technical procedures between laboratories and genetic differences among populations or individuals can also contribute to variation in expression divergence.

In theory, if the “target” species for which the array was designed and a “non-target” species are closely related, some probes on the array should be able to interrogate expression of genes in both species without bias if the sequences that are interrogated by the probes are still the same in both species [11]–[13]. Some studies have attempted to identify and eliminate probes with biased response to the transcriptome of the target and non-target species. One tactic is to select probes on the basis of genomic DNA (gDNA) hybridizations of the target and a non-target species to the microarray chip [10], [14], [15]. If the same amount of gDNA is used in the hybridization, probes that match conserved regions should hybridize with similar intensity to gDNA in both species. Recently, for example, the *Xenopus laevis* Affymetrix microarray chip was used to explore expression divergence between different species of clawed frogs and their hybrids [16]–[18]. Comparisons were made between testis and ovary expression profiles of the target species, *X. laevis* (XL), a non-target species, *X. muelleri* (XM), and F1 hybrids from a cross between a XL female and a XM male (hereafter H_XLXM_). In these studies, hybridizations of gDNA of XM and XL were performed on the XL microarray, and probes whose XM/XL genomic hybridization intensity ratio (gDNA ratio) was not between 0.99 and 1.01 [17] or between 0.99 and 1.10 were excluded from the analysis [17], [18]. When expression profiles of testes or ovaries of XL and XM were compared to the same tissue in their hybrids, widespread dominance in expression was reported in hybrids wherein the expression profile of H_XLXM_ tended to be more similar to XL than to the non-target parental species XM [17], [18]. About 28 times more genes were significantly divergently expressed in testes in a comparison between XM and H_XLXM_ than between XL and H_XLXM_ [17] and about 4.5 times more genes were significantly divergently expressed in ovary in a comparison between XM and H_XLXM_ than between XL and H_XLXM_ [18].

With a goal of further exploring these results, we analyzed new expression data from testis tissue of XL, *X. borealis* (XB), and F1 hybrids between XL x XB (XL female and XB male, hereafter H_XLXB_). XB and XM are equivalently diverged from XL [19]–[21] so our new data provide a phylogenetically meaningful comparison. All of these species are “pseudotetraploid” in that they are historically tetraploid but their genomes have diploidized (bivalents form at meiosis; each chromosome has only one homologous chromosome). XL and (XB+XM) diverged from a common tetraploid ancestor roughly 21–41 million years ago, and XB and XM diverged from a common ancestor roughly 14–25 million years ago [19]–[22]. In the analysis of these new expression data, we included only those probes that interrogate sequences that are identical in XL and in XB based on 454 pyrosequencing of XB cDNA. For comparative purposes, we also performed genomic DNA hybridizations on XL, XB, and XM, and analyzed the new data and also data from other studies [16]–[18], [23], [24] using microarray probes selected using the gDNA hybridization approach of [17], [18], [23].

## Results

### Affymetrix *Xenopus laevis* microarray, probemasks, and tissue comparisons

This study examines expression data collected from a prefabricated *Xenopus laevis* microarray – the Affymetrix GeneChip® *Xenopus laevis* Genome Array. This microarray interrogates over 14400 transcripts. A transcript is interrogated with a set of 16 probes, which is called a “probeset”. Each probe within a probeset is an oligonucleotide 25 base pairs in length that hybridizes to a unique portion of an XL transcript. For each species or hybrid in this study, three biological replicates (different individuals) were performed per tissue. Hereafter we refer to the replicated expression data from a single tissue from one species or one type of hybrid (either H_XLXB_ or H_XLXM_) as a “treatment”.

Probemasks are lists of genes that are defined *a priori* to be excluded from analysis (before microarray normalization is performed). In this study, we analyzed data using two types of probemasks. The first type of probemask excluded all probes except those that interrogated sequences that we confirmed were identical in XL and XB, as in [9], [25]. We used BLAST [26] to identify probes on the Affymetrix GeneChip® *Xenopus laevis* Genome Array that perfectly match sequences in XB that we obtained using 454 pyrosequencing of normalized XB testis cDNA. Normalization of XB testis cDNA (which is a procedure different from and unrelated to normalization of microarray data) was performed prior to 454 pyrosequencing in order to increase representation of genes with low expression; procedures for cDNA normalization and 454 pyrosequencing are described elsewhere [27]. The resulting probemask included 5268 probes in a total of 2143 probesets, for an average of 2.458 probes per probeset. Hereafter we refer to this probemask as the “XB+XL perfect match probemask”. According to a permutation test in which the same number of probes is assigned to probesets randomly one thousand times, this average number of probes per probeset is significantly higher than random expectations (P<0.001; the mean number of probes per probeset of the permutations was 1.169 and the 95% confidence interval was 1.158–1.180). This is consistent with the notion that some genes are conserved across multiple regions that are interrogated by unique probes on the microarray, resulting in significantly more probes per probeset than random expectations. Despite this biologically relevant pattern, we note that the overall low number of probes per probeset is likely to be associated with more variation in expression intensities than is typical of Affymetrix probesets with 16 probes. Furthermore, because the perfect match probes identified in XB are based on 454 pyrosequencing of normalized testis cDNA, this analysis might be biased in favor of genes that are expressed in testis of this non-target species. Additionally, because we retain only those probes that are identical in XL and XB, this analysis probably is also biased towards slowly evolving genes – or at least genes that have slowly evolving regions that are interrogated by probes on the microarray.

The second type of probemask was generated based on the non-target to target hybridization ratio of genomic DNA (the gDNA ratio) of XL, XB, and XM as in [17], [18]. These probemasks include only those probes with a non-target/target gDNA ratio between 0.99 and 1.1, and hereafter we refer to them as the “XB/XL gDNA probemask” and the “XM/XL gDNA probemask”, respectively. The XB/XL gDNA probemask included a total of 1792 probes in 1672 probesets, for an average of 1.072 probes per probeset. This average is similar but still significantly higher (P = 0.003) than random expectations according to a permutation test, which had an average of 1.055 (95% confidence interval 1.045–1.067). This average is significantly lower than the average of the XB+XL perfect match probemask (P<0.001, permutation test). Only 2.5% of the probes (45 out of 1792 probes) that were retained by the XB/XL gDNA probemask are also retained by the XB+XL perfect match probemask. Less than 1% of the probes (45 out of 5268 probes) that were retained by the XB+XL perfect match probemask were also retained by the XB/XL gDNA probemask.

The XM/XL gDNA probemask included a total of 12888 probes in 8721 probesets and an average of 1.478 probes per probeset. This average is also similar but still significantly higher than the corresponding average of the random permutations of 1.448 (P<0.001; 95% confidence interval 1.437–1.460). For comparison, the probemask of [17] included 11485 probesets with an average of less than 2 probes per probeset.

Using both types of probemask (the XB+XL perfect match probemask and the XB/XL gDNA probemask), we evaluated interspecific expression divergence in testis between H_XLXB_ and each parental species (XL or XB) and in brain between XL and XB. We also used both of these probemasks to evaluate intraspecific expression divergence between various XL tissues (egg, tadpole stage 11, ovary, testis, and brain). Additionally, we used the XM/XL gDNA probemask to evaluate expression divergence in testis and ovary between H_XLXM_ and each parental species (XL or XM) and we used this same probemask to evaluate intraspecific expression divergence between the aforementioned XL tissues. We were not able to perform interspecific analyses between XL and XM with a perfect match probemask because sequence data from XM was not obtained.

### Dominant expression in hybrids?

When we analyzed testis expression data from XL, XB, and H_XLXB_ using the XB+XL perfect match probemask, expression divergence between XL and H_XLXB_ is slightly less than between XB and H_XLXB_, but similar in terms of the number of significantly divergently expressed genes. Out of 2143 probesets included in this analysis, 182 genes are significantly upregulated in XL testis compared to H_XLXB_ testis whereas 210 genes are significantly upregulated in H_XLXB_ testis compared to XL testis. 280 genes are significantly upregulated in XB testis compared to H_XLXB_ testis whereas 245 genes are significantly upregulated in H_XLXB_ testis compared to XB testis. The number of significantly upregulated genes in each parental species compared to H_XLXB_ is significantly higher in the comparison with XB than the comparison with XL (182 versus 280, G = 20.95, P<0.001, two-sided test). But the number of significantly upregulated genes in H_XLXB_ compared to each parental species is not significantly different (210 versus 245, G = 2.69, P = 0.20, two-sided test). Therefore, the difference in the number of significantly divergently expressed genes in each comparison between a parental species and hybrids is attributable to more genes being upregulated in XB compared to H_XLXB_ than are upregulated in XL compared to H_XLXB_. Thus, the proportion of divergently expressed genes in XB testis compared to H_XLXB_ testis is about 1.34 times as large as the proportion of divergently expressed genes in XL testis compared to H_XLXB_ testis (Table 1). But, as mentioned earlier, some or all of this bias could be because we retained probes in this analysis based on sequences of genes that are expressed in XB testis.

**Table 1:** Proportions of divergently expressed genes differ significantly depending on what probemask is used in the analysis.

	XL versus H			NT versus H				
Analysis	SUXL	SUH	Proportion divergently expressed	SUNT	SUH	Proportion divergently expressed	Ratio of divergently expressed genes	Number of genes analyzed
XB + XL perfect match probemask	182	210	0.18	280	245	0.24	1.34	2143
XB/XL gDNA probemask	79	97	0.11	468	299	0.46	4.36	1672
XM/XL gDNA probemask	417	572	0.11	1430	1248	0.31	2.71	8721
Malone et al. (2007)	92	50	0.01	2236	1759	0.35	28.13	11485
Malone et al. (2008)	777	839	0.14	4349	2930	0.63	4.50	11485

Results are shown from pairwise comparisons between XL and H (XL versus H) and between a non-target species and a hybrid (NT versus H). All analyses compare testis tissue except the ones from [18], which compare ovary tissue. For each comparison, the number of significantly upregulated genes in XL (SUXL), significantly upregulated genes in the hybrid (SUH), and significantly upregulated genes in the non-target species (SUNT) is listed. The proportion of divergently expressed genes is equal to the total from the (NT versus H) comparison divided by the total from the (XL versus H) comparison.

While this 1.34 fold asymmetry in divergent expression between the parental species and hybrids is significant (525 versus 392 genes, G = 19.36, df = 1, P<0.001), it is in sharp contrast with the 28 fold difference reported in comparisons between testis tissue of XL, XM, and H_XLXM_ where 3995 genes were divergently expressed between XM and H_XLXM_ but only 142 genes were divergently expressed between XL and H_XLXM_ [Table 1; 17]. The difference in the proportion of divergently expressed genes in this study compared to [17] is significant. More specifically, a re-sampling test (see Methods) indicates that there is a significantly higher proportion of divergently expressed genes between XL and H_XLXB_ using the XB+XL perfect match probemask than were reported between XL and H_XLXM_ by [17] using a gDNA probemask (P<0.001). Likewise, there is a significantly lower proportion of divergently expressed genes between XB and H_XLXB_ using the XB+XL perfect match probemask than were reported between XM and H_XLXM_ by [17] (P<0.001).

With respect to misexpression – which we define as hybrid expression that is not intermediate with respect to the expression of each parental species – using the XB+XL perfect match probemask, we find that only 13 genes are significantly upregulated in testis of H_XLXB_ with respect to testis of both XL and to XB and that 16 genes are significantly upregulated in testis of XL and XB with respect to testis of H_XLXB_. This difference is not significant (G = 0.31, df = 1, P = 0.58).

### Comparison of gDNA hybridizations within and between species

To further explore the basis of the discrepancy in the level of asymmetry of divergent expression recovered by our results using the XB+XL probemask and previous studies, we re-analyzed testis expression data from XL, XB, and H_XLXB_ using the XB/XL gDNA probemask that was based on our new gDNA hybridizations. We also re-analyzed testis and ovary expression data from XL, XM, and H_XLXM_ using the XM/XL gDNA probemask that was based on our new gDNA hybridizations.

**Figure 1:**
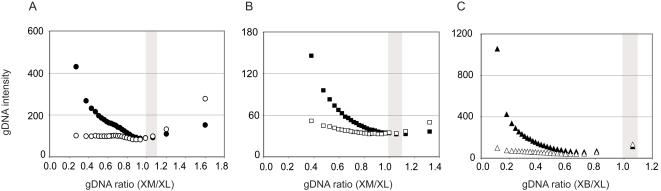
Genomic hybridization intensities (gDNA intensity) of XL, XB, and XM vary with respect to the non-target to target ratio of these intensities (gDNA ratio). This graph depicts the median gDNA intensities of all probes on the chip ranked by their gDNA ratio into 25 bins; each bin contains 10,000 probes except the 25^th^ bin, which contains 7852 probes. The area in gray corresponds with the range of gDNA ratios of probes that are retained using the method of Malone et al. (2007). XL gDNA ratios are represented by filled symbols and non-target gDNA ratios are represented by unfilled symbols. Shown are relationships between the median gDNA intensity of each bin and the median gDNA ratio of each bin for (A) our XM and XL gDNA hybridizations, (B) the XM and XL gDNA hybridizations of Malone et al. (2007), and (C) our XB and XL gDNA hybridizations.

We compared our gDNA hybridizations to those of [17], [18]. We ranked all of the probes on the chip by the gDNA hybridization intensity and then divided these ranks into 25 bins. Comparison to the gDNA ratio of each probe indicates that the median intensity of hybridization was lower in the non-target species (XM or XB) than the target species (XL) for most bins (Fig. 1). Probes with a gDNA ratio near one tended to have lower gDNA hybridization intensities in both the non-target and the target species than other probes on the chip, and the target species (XL) tends to have a more dynamic relationship between probe intensity and the gDNA ratio. Thus, at least on the Affymetrix GeneChip® *Xenopus laevis* Genome Array, probe selection on the basis of a gDNA hybridization ratio near one appears to have an unintended consequence of retaining probes with low gDNA hybridization intensities in both species. This was true in gDNA hybridizations performed by our lab and also by another lab (Fig. 1), thus it is not attributable to differences in laboratory procedure.

Our XB gDNA hybridization was less intense than our XM hybridization even though we attempted to control for the amount of gDNA used in the hybridization, and even though these species are equally diverged from XL (Fig. 1). This variation probably is technical in nature and underscores the challenge of generating comparable gDNA hybridizations for different species. Below we report results derived from analyses based on our gDNA hybridizations for XL, XB, and XM; as detailed below, these results are qualitatively similar to those recovered with the gDNA probemask of [17], [18].

### Is the ratio of genomic DNA hybridization a reliable way to detect perfect match probes on the *Xenopus laevis* Affymetrix chip?

**Figure 2:**
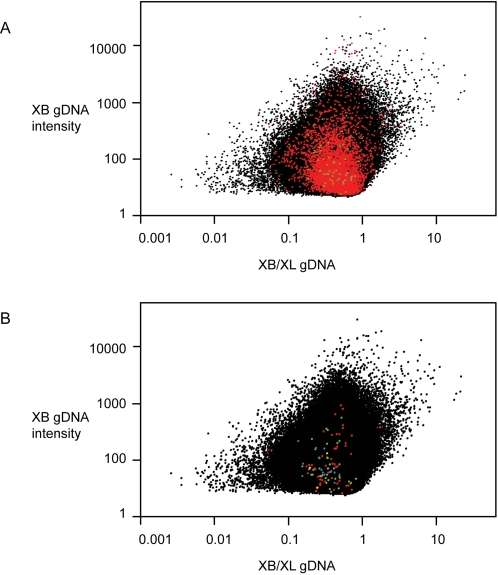
The gDNA ratio of probes that perfectly match (PM) XL and XB overlaps extensively with the gDNA ratio of probes that mismatch (MM) one species. (A) XB gDNA intensity versus gDNA ratio of PM probes in XL, XL and XB, and XB. PM probes in XL are in black, PM probes in XL and XB are in red, and PM probes in XB but not XL are in green. (B) XB gDNA intensity versus gDNA ratio of MM probes in XB. For comparative purposes, PM probes in XL are again in black. Probes that mismatch both paralogs of genes in XB with one, two, three, or four base pair differences are indicated in red, blue, green, and yellow respectively.

Probes that perfectly match sequences from XL and XB have a wide range of XB/XL gDNA ratios (Fig. 2A). Under a best-case scenario, this indicates that using the gDNA ratio as a criterion for probe retention will not retain all perfect match probes. But we also found that other probes that we know mismatch both paralogs of genes in XB have a range of XB/XL gDNA ratios that overlaps extensively with the gDNA ratios of probes that perfectly match both species (Fig. 2B). This point is also illustrated by examination of four probesets on the *Xenopus laevis* Affymetrix microarray that were designed to interrogate XB transcripts: XlAffx.1.5.S1_at, XlAffx.1.9.S1_at, XlAffx.1.10.S1_at, and XlAffx.1.12.S1_at. Sixty out of the 64 probes in these four probesets do not perfectly match XL, and these also have a broad range of gDNA ratios (Fig. 2A). Together these observations indicate that gDNA ratios provide a poor basis for selection of perfect match probes in non-target species on the Affymetrix GeneChip® *Xenopus laevis* Genome Array. In addition to not retaining many probes that perfectly match both species, this approach almost certainly results in the retention of probes that do not perfectly match the non-target species.

### Does it matter if some probes with differential performance between treatments are included in the analysis?

When testis expression data from XL, XB, and H_XLXB_ are analyzed using our XB/XL gDNA probemask or using our XM/XL gDNA probemask, we recover similar results to the analysis of testis expression data from XL, XM, and H_XLXM_ by Malone et al. [17]. This suggests that evolutionary differences between XB and XM, possible differences in the geographic origin of XL animals, and variation in laboratory procedures associated with microarray hybridizations together had a much smaller impact on the results than did the type of probe mask used in the analysis. More specifically, in this analysis the asymmetry in expression divergence is significant and more substantial than results from the XB+XL perfect match probemask such that expression in the hybrid appears much more similar to the target than the non-target species (Table 1). This is because using a gDNA probemask instead of a perfect match probemask results in a significantly lower proportion of genes that are divergently expressed in the comparison between XL and H_XLXB_ and a significantly higher proportion of genes that are divergently expressed between XB and H_XLXB_ (P≤0.002 for both comparisons).

We explored alternative analytical approaches including invariant set (IS) normalization [28] and the probe logarithmic intensity error (PLIER) method for calculating signal intensity [29]. These procedures produce results that are qualitatively similar to those recovered with RMA normalization with each probemask. The asymmetry in divergent expression in testis between each parental species and the hybrid with the XB+XL perfect match probemask is of similar magnitude in each of these analyses (1.34, 1.45 and 1.39 for RMA, IS, and PLIER, respectively). Likewise, more than twice as much asymmetry in divergent expression in testis is recovered when RMA, IS, or PLIER normalization are used with gDNA probemasks (i.e. there are more divergently expressed genes between the non-target species and the hybrid than between XL and the hybrid with these probemasks; data not shown). Thus we conclude that the method of normalization also does not account for the substantial differences in results that are obtained from perfect match versus gDNA probemasks.

### Rank difference

The nature of the discrepancy between results obtained from these different probemasks is further illuminated by consideration of some of the technical aspects of the analysis. When microarray data are normalized it is generally assumed that the overall distribution of expression intensities within each treatment is similar [30]–[32]. Moreover, most normalization methods were developed for comparisons between treatments with expression divergence at only a few genes [33]. When data are normalized with the quantile method [30], for example, which was used in this study and in [16]–[18], the expression intensities of each probe are ranked and replaced by the average intensity of each quantile (each rank). This procedure yields identical distributions of overall expression intensities across treatments, even if they were very different to begin with.

**Figure 3:**
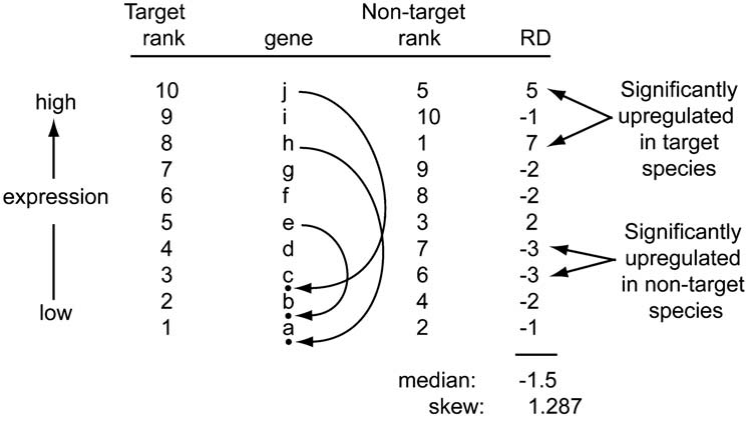
An example of how poor performance of a few probes in the non-target species can affect the rank of many genes, even ones that perform equally in both species. Ten genes (a, b, c, d, e, f, g, h, i, and j) are ranked according to their expression intensity. In the non-target species, probes directed against genes e, h, and j perform poorly and have a low rank in the non-target species due to sequence divergence, even though there actually is no expression divergence. This elevates the rank of many other genes, causing an overall negative median rank difference (RD) and a positively skew in the RD distribution. In this example, significantly upregulated genes in the target species tend to have a higher average rank in this species (9) than the significantly upregulated genes in the non-target species do in that species (6.5). Significantly upregulated genes in the target species have a lower average rank in the non-target species (3) than the significantly upregulated genes in the non-target species do in the target species (3.5).

If the overall distribution of expression intensities was similar in each treatment before normalization, it is reasonable to expect that the magnitude and direction of expression divergence should be unbiased – that for a given magnitude of expression divergence, a similar number of genes will be upregulated in one treatment as is upregulated in the other. To test this, we calculated the difference in expression rank for each gene included in the analysis, with the lowest rank corresponding to the gene with the lowest expression as depicted in Fig. 3. Additionally, the skew of this distribution was quantified by the Pearson skewness coefficient ( = 3*(mean-median)/standard deviation). Departure of the observed median rank difference and skew of the distribution of rank differences from the null hypotheses of a median and skew of zero was assessed by comparison to a null distribution generated from 1000 randomized ranks using scripts written in PERL.

When interspecific data from the target species and a non-target species were analyzed using a gDNA probemask, the median rank difference was negative and this median departed significantly and substantially from zero (Table 2). The skew of the distribution of rank differences was significantly and substantially positive in these interspecific comparisons (Table 2). While these metrics are not independent because the median is used in the calculation of skew, they provide qualitative information about the rank difference distributions in these analyses. Because we calculated the rank difference by subtracting the non-target rank from the target rank, a negative median indicates that the non-target sequences tend to be upregulated to a greater degree than do the target sequences. A positive skew of this distribution (Table 2) indicates a tail on the right, suggesting that some probesets have a much higher rank (higher expression) in XL but not the reverse.

**Table 2:** Analyses with gDNA probemasks produce different rank difference distributions in interspecific and intraspecific comparisons.

Comparisons with XB/XL gDNA probemask^a^			
Interspecific comparisons	Median	skew	δSRC
XL_T_-XB_T_	−54*	0.409*	0.2837^Χ^
XL_B_-XB_B_	−58*	0.476*	0.1811^Χ^
XL_T_-XB_B_	−51*	0.376*	0.1595^Χ^
XL_B_-XB_T_	−54.5*	0.391*	0.2231^Χ^
Hybrid to parental comparisons			
XL_T_-H(XLXB)_T_	−1	0.016	0.0245
H(XLXB)_T_-XB_T_	−51	0.470	0.2986^Χ^
Intraspecific comparisons			
XL_T__XL_E_	−16	0.140*	0.0794
XL_T_-XL_T11_	−0.5	0.004	0.0304
XL_T_-XL_O_	17	−0.161*	0.0154
XL_O_-XL_T11_	0	0.000	0.0446
XL_O__XL_E_	−12	0.166*	0.0578
XL_E__XL_T11_	14	−0.178*	0.1325
XL_T__XL_B_	12	−0.114*	0.0689
XL_B__XL_E_	−13	0.096	0.1578
XL_B__XL_o_	11	−0.079	0.0274
XL_B__XL_T11_	2.5	−0.018	0.1608
**Comparisons with XM/XL gDNA probemask^b^**			
Interspecific comparisons			
XL_T_-XM_T_	−269*	0.417*	0.2153^Χ^
XL_O_-XM_O_	−390*	0.568*	0.2501^Χ^
XL_T_-XM_O_	−250*	0.374*	0.1335^Χ^
XL_O_-XM_T_	−338*	0.429*	0.1906^Χ^
Hybrid to parental comparisons			
XL_T_-H(XLXM)_T_	11	−0.032	0.0129
H(XLXM)_T_-XM_T_	−151*	0.257*	0.1810^Χ^
XL_O_-H(XLXM)_O_	−32	0.092*	0.0077
H(XLXM)_O_-XM_O_	−187*	0.309*	0.1642^Χ^
Intraspecific comparisons			
XL_T__XL_E_	14	−0.024	0.0091
XL_O_-XL_T11_	14	−0.031	0.0642
XL_T__XL_0_	109*	−0.198*	0.0535
XL_T__XL_T11_	48	−0.081*	0.0043
XL_O_-XL_E_	−67*	0.175*	0.0477
XL_E_-XL_T11_	77*	−0.192*	0.1313
XL_T__XL_B_	17	−0.031	0.0639
XL_B__XL_E_	−6	0.009	0.0598
XL_B__XL_O_	69*	−0.095*	0.0278
XL_B__XL_T11_	51	−0.074*	0.0749

Median and skew of the rank difference distribution and δSRC (see text) are reported. Suffixes after species acronyms (XL, XB) refer to the tissue type analyzed: O (ovary), T (testes), T11 (tadpole stage 11), B (brain), and E (egg). Asterisks indicate significant departure from the null. For δSRC, interspecific comparisons and comparisons between a non-target species and a hybrid are higher than other comparisons, and are indicated (^Χ^). In all of these cases, the correlation (i) is higher than the correlation (ii).

a 1672 probesets, CI median: 0±24, CI skew: 0±0.107

b 8721 probesets, CI median: 0±54, CI skew: 0±0.046

In contrast, when intraspecific comparisons were analyzed with gDNA probemasks, the median and skew never departed as substantially from the null expectation as the interspecific comparisons between a target and non-target species, although occasionally the intraspecific departure was significant (Table 2). When the XB+XL perfect match probemask was used in the analysis, the median and skew were not significantly different from the null expectation (Table 3). While occasional departure from the null in some intraspecific comparisons between different XL tissues probably has a biological basis and could also stem from variation between laboratories in microarray protocol, these comparisons suggest that the substantially negative median and positive skew of the rank difference in interspecies comparisons analyzed with gDNA probemasks has a technical rather than a biological basis.

**Table 3:** Analysis with the XB+XL perfect match probemasks produces results with similar rank difference statistics in interspecific and intraspecific comparisons.

Comparisons with XB+XL perfect match probemask^a^			
Interspecific comparisons	median	skew	δSRC
XL_T_-XB_T_	1	−0.009	0.0029
XL_B_-XB_B_	1	−0.014	0.0094
XL_T_-XB_B_	4	−0.035	0.0076
XL_B_-XB_T_	1	−0.007	0.0045
Hybrid to parental comparisons			
XL_T_-H(XLXB)_T_	4	−0.050	0.0167
XB_T_-H(XLXB)_T_	−2	0.031	0.0197
Intraspecific comparisons			
XL_T__XL_E_	−22	0.130*	0.0946
XL_T_-XL_T11_	−13	0.079	0.0558
XL_T_-XL_O_	−11	0.072	0.0513
XL_O_-XL_T11_	−2	0.018	0.0174
XL_O__XL_E_	−11	0.113*	0.0343
XL_E__XL_T11_	3	−0.030	0.0408
XL_T__XL_B_	4	−0.027	0.0008
XL_B__XL_E_	−29*	0.154*	0.0619
XL_B__XL_o_	−10	0.052	0.0109
XL_B__XL_T11_	−20	0.110*	0.0710

Acronyms and statistics follow Table 2.

a 2143 probesets, 95% confidence interval (CI) of the median = 0±25, and CI of the skew = 0.000±0.087

### Spearman rank correlation

When gDNA probemasks are used, we suspected that differential performance of some probesets in the non-target species could cause a spurious signal of upregulation *and* downregulation compared to another species (Fig. 3). One class of significantly differently expressed genes – those that appear to be upregulated in the target species (XL) – could result when probes hybridize poorly to transcripts of the non-target species. The other class of significantly differently expressed genes – those that appear to be upregulated in the non-target species (XB or XM) – could result when the ranks of some genes in the non-target species are elevated as a result of the other genes that are interrogated by biased probes having a lower rank (Fig. 3). A key difference between these two classes of divergently expressed genes is that a larger proportion of the genes that appear upregulated in XL are interrogated by probes with differential performance (bias) between species. In analyses with a gDNA probemask, therefore, we predicted that the expression rank of genes that appear to be significantly upregulated in the non-target species would be highly correlated with the expression rank of these genes in the target species. We expected this correlation to be much higher than the correlation between the ranks of genes upregulated in the target species and the rank of these same genes in the non-target species.

To test this, we calculated the Spearman's rank correlation (SRC) of the rank in each treatment of (i) genes upregulated in the non-target species and (ii) genes upregulated in the target species. Under our hypothesis that many of the genes that are upregulated in the non-target species are false positives, we expected that the SRC would be much higher in (i) than in (ii). To quantify this expectation, we calculated the absolute value of the difference in the SRC in (i) and (ii) for the interspecies comparisons, and we refer to this difference as δSRC. For comparative purposes, δSRC was calculated for interspecific comparisons between XL and a non-target species, comparisons between each species and a hybrid, and intraspecific comparisons between different tissues of XL, and this was performed for analyses with each type of probemask.

The data support our expectation. When the XB/XL gDNA probemask or the XM/XL gDNA probemask are used in interspecific comparisons, the δSRC of the rank of genes upregulated in the non-target species is substantially higher than that of genes upregulated in the target species or in hybrids (Table 2). When comparisons were made between tissue types in XL or within a tissue type of XL and a hybrid using these gDNA probemasks, extreme differences between δSRC of each of these classes of genes were not observed (Table 2). A high δSRC was not observed in any of the analyses with the XB+XL perfect match probemask (Table 3). Furthermore, we found other signs of technical bias in results generated with gDNA probemasks, but not the XB+XL perfect match probemask, by comparing the mean rank of significantly upregulated genes (Supporting Information S1, Table S1, Table S2).

Taken together, these observations are consistent with the notion that the use of probemasks based on gDNA ratios on the Affymetrix GeneChip® *Xenopus laevis* Genome Array produces spurious results when comparisons are made directly between species or between a non-target species and a hybrid, irrespective of tissue type. When gDNA probemasks are used, many of the genes that are putatively upregulated in the non-target species are actually false positives whose high ranks are an artifact of the low ranks of poorly performing probesets. Of course, this group of genes may include some genes that are not false positives, but it is not clear which ones these are. We suspect then, albeit with caveats discussed below, that our analysis with the XB+XL perfect match probemask is a closer approximation of biological variation than that recovered by [17], [18].

## Discussion

### Probe selection by genomic hybridization

A challenge to the implementation of single-species microarrays in comparative transcriptomics is the identification of unbiased probes. Due to differences from the target species, such as sequence divergence, non-target transcripts will exhibit a range of probe hybridization efficiencies that cause technical variation in hybridization intensities. In comparative analyses, normalization may overcompensate for genes with lower than average divergence and undercompensate for genes with higher than average divergence [34]. Exacerbating this problem, our analysis of confirmed perfect match probes in a target and a non-target species illustrates that the gDNA ratio is an unreliable metric with which to identify unbiased probes on the Affymetrix GeneChip® *Xenopus laevis* Genome Array. This approach selects probes with low gDNA intensity (Fig. 1), misses probes that do perfectly match both species (Fig. 2A), and includes probes that do not perfectly match both species (Fig. 2B). The implications of this are large and affect fundamental conclusions of the analysis, such as which and how many genes are significantly or not significantly differently expressed. Notably, our analyses suggest that including biased probes in a microarray analysis leads not only to spurious results from these biased probes, but affects conclusions drawn from probes that are interrogated by probes that perform equally well in both species. We anticipate, therefore, that comparisons between species using probes that are selected by gDNA ratios, including the comparison between XB or XM and XL that are presented here, are characterized by a high level of false positives as well as false negatives. Many of the genes from this type of analysis that are putatively upregulated in the target species are actually interrogated by probes that do not perform equivalently in the non-target species. Many of the genes that are putatively upregulated in the non-target species are actually genes whose ranks have been elevated as an artifact of other probes that do not perform equivalently in the non-target species. It is therefore not only necessary to retain as many perfect match probes as possible, but also to exclude biased probes from microarray analyses.

### Gene duplication

Another concern with the application of this microarray to non-target clawed frog species relates to whole genome duplication. Because XL, XB and XM are tetraploid, asymmetry in cross-hybridization between paralogous transcripts could influence results. For example, a probe might hybridize to only one paralog in one species but to both paralogs of genes in another species, either as a result of sequence divergence or because both are expressed in one but not the other. This problem is aggravated by species-specific pseudogenization. Estimates of the percent of duplicated genes in XL that are still expressed (not pseudogenes) range from 77% [35] to a probably more accurate estimate of less than 50% [36], [37]. Divergence of the ancestor of XL from the ancestor of (XM+XB) occurred about halfway between the time of whole genome duplication and the present [19], [22], [27]. For this reason, the frequency of expressed orthologous transcripts in XL and non-target species such as XB and XM is far below 100% as a result of “divergent resolution” – the retention of different (non-orthologous) paralogs of genes in each species [38].

That Affymetrix microarrays do not effectively discriminate between different but closely related duplicated genes has been suggested for allopolyploid wheat [39]. However, we performed a power analysis that indicated that probes on the XL microarray performed consistently in distinguishing expression of each paralog after the application of probemasks with different specificities for a target paralog [i.e. varying numbers of mismatches to the non-target paralog; 27]. But within *Xenopus*, orthologs are more similar to each other than are paralogs derived from genome duplication because genome duplication occurred before speciation. Orthologous but not identical sequences (from different species) thus have greater potential to be able to hybridize strongly but not equivalently to probes directed against one XL paralog, than do co-expressed paralogs within XL. These concerns are relevant to all of the analyses presented here, including the ones that use the XB+XL perfect match probemask.

### Conclusions

Previous work has explored factors in addition to sequence divergence that influence probe hybridization efficiency in different species, such as variation in labeling, overlap of oligonucleotide probes, alternative splicing, sequence homology to non-target transcripts, insertion/deletion differences, and intraspecific polymorphism [33], [34], [39]–[43]. Some or all of these variables might be at play here – sequence divergence, for example, has already been shown to influence microarray hybridization efficiency in clawed frogs [44]. While sequence mismatches might not substantially affect the ability of microarrays to detect misexpression [45], [46], it seems probable that sequence mismatches could cause bias if it varies among treatments such as when expression of two species are compared using an array designed for only one of them. Therefore, an experimental design that has consistent bias across treatments, in which one compares ‘apples to apples’ [47], has the potential to provide useful information from non-target species. Examples of more appropriate experimental designs include (a) using a microarray designed for another species with a non-target species but only comparing intraspecific expression levels within the non-target species, (b) constructing custom arrays for each species (or hybrid) of interest, and (c) building a custom array with probes directed against each species [46]. Another important measure for comparative analyses using single-species arrays is the validation of results using microarray-independent approaches, such as real-time quantitative PCR. The biases suggested by our analyses have implications for studies that deploy Affymetrix microarrays for interspecific comparisons, particularly [16]–[18], and could also be a concern for expression studies of species or genes with population structure, high mutation rate, or large effective population size.

## Materials and Methods

### Origin of animals

XB expression data, gDNA, and XB parents of H_XLXB_ were from or were animals from Kenya. The XL expression data, gDNA, and XL parents of H_XLXB_ were from or were laboratory animals that probably are from Cape Province, South Africa, which is the source of most laboratory stocks [48]. All of the H_XLXB_ individuals were from the same cross and are therefore full siblings. We did not analyze hybrid tissue from the reciprocal cross (from an XB female and XL male).

The XM expression data, gDNA, and parents of H_XLXM_ in [17], [18] were or were from animals collected in Swaziland, but the XM gDNA that we performed for gDNA hybridization originated from Tanzania. Within XM, mitochondrial DNA variation between these localities is very low so we do not anticipate as substantial levels of intraspecific variation in the nuclear genome of this species compared to XL [19].

### Microarray hybridizations and comparisons

We performed new expression analyses on testis and brain tissue from XL, XB, and H_XLXB_. For each tissue from each species or hybrid, RNA was isolated using TRIzol® Reagent (Invitrogen Life Technologies) according to the manufacturer's protocol, purified with RNeasy Mini Kit (Qiagen), and its integrity assessed on an Agilent BioAnalyzer. Two micrograms of total RNA was used to prepare biotin labeled cRNA probes, which were subsequently hybridized to Affymetrix *Xenopus laevis* expression arrays following the manufacturer's protocol.

We performed new gDNA hybridizations using gDNA from XL, XB, and XM and compared these to gDNA hybridizations on XL and XM that were performed by Malone et al. [17]. For our gDNA hybridizations, five micrograms of gDNA from each species was fragmented with Dpn I at 37°C for 3 hours. Fragmented gDNA was purified with Qiagen PCR clean-up kit and the fragment distribution was checked on Agilent Bioanalyzer (Agilent) using the DNA 1000 assay. 50–100 nanograms of fragmented gDNA were then amplified using the BioPrime Labeling System (Invitrogen) following the manufacturers instructions. After completion of the Klenow Pol I catalyzed reaction, the distribution of PCR products was examined on Agilent Bioanalyzer with the DNA 1000 kit. The entire volume of the product (∼50 μl) was used in the hybridization reaction on the Affymetrix *Xenopus laevis* Gene Chip. Hybridization, staining, washing and scanning were performed as described in the Expression Analysis Technical Manual. This protocol is similar to that used by Hammond et al. [14].

After scanning, raw expression data were converted into CEL files using Microarray Analysis Suite version 5 (MAS 5, Affymetrix). For each pairwise comparison, CEL files were pre-normalized with the Robust Multichip Average (RMA) algorithm in RMAexpress [49] using custom CDF files (probemasks) and the default parameters, which include a median polish and quantile normalization. The normalized data were used in the R statistical package following the protocol in [17]. An empirical Bayesian model was used to compute a moderated t-statistic using the limma package from Bioconductor [50]. The TopTable function gave a P-value for differential expression for each gene that was adjusted using the Benjamini and Hochberg [51] method to control for the false discovery rate. cDNA and gDNA hybridizations that we performed have been deposited in the Gene expression omnibus database [52], GEO Series accession number GSE12625. We also analyzed other data from this database (GSM241082-4 [23], GSM99995-7 [24], GSM99980-2 [24]). Expression data and genomic hybridizations from XL and XM testis and ovary that were not found in GEO were kindly provided by Pawel Michalak.

We used a re-sampling approach to test whether the proportion of divergently expressed genes in different analyses (each with a unique number of genes analyzed) were significantly different. Given two analyses with w and x genes of which y and z are significantly divergently expressed, respectively, using a PERL script we generated 1000 simulated datasets, each with w genes, by re-sampling a distribution of (w+x) total genes with (y+z) genes that are significantly divergently expressed. Where (y/w)<(z/x), the two-sided probability of the null hypothesis of no difference is twice the proportion of these simulated datasets that had a proportion of divergently expressed genes lower than y/w (i.e. more different from z/x). Because some of the genes in these different analyses are the same and should therefore have correlated expression levels, the inclusion of these genes in this comparison reduces the power to reject the null hypothesis, making this test conservative.

## Supporting Information

Supporting Information S1 Inspection of mean rank of significantly upregulated genes provides additional support for bias in gDNA probemasks.
(0.05 MB DOC)

Table S1 Mean ranks of significantly upregulated genes when analyzed with gDNA probemasks.
(0.03 MB XLS)

Table S2 Mean ranks of significantly upregulated genes from analysis using the XB+XL perfect match probemask.
(0.02 MB XLS)
