# Supplementary material for: Single-Species Microarrays and Comparative Transcriptomics
Source: PLoS One. 2008 Sep 25;3(9):e3279. doi: 10.1371/journal.pone.0003279 (PMC2533705; doi:10.1371/journal.pone.0003279)
Supplement: Supporting Information S1 — Inspection of mean rank of significantly upregulated genes provides additional support for bias in gDNA probemasks. (0.05 MB DOC) [file pone.0003279.s001.doc]

**SUPPORTING INFORMATION**

Supporting Information 1: Additional support for pervasive bias in analyses with gDNA probemasks.

That technical bias influences which genes are significantly upregulated is also evinced by inspection of the ranks of significantly upregulated genes in XL and the non-target species (NT) using different probemasks. In comparisons between species using gDNA probemasks, the mean rank in XL of the significantly upregulated genes in XL is always higher than the mean rank in NT of the significantly upregulated genes in NT (Table S1). As depicted in Figure 3, this is consistent with some of the upregulated genes in XL being upregulated because poor probe performance in NT. In other words, if the rank of a gene in XL is high, then the difference between the rank of that gene in XL and NT can be more substantial (and therefore significant) if probes for that gene perform poorly in NT. In these same comparisons, the mean rank in XL of the significantly upregulated genes in NT is always higher than the mean rank in NT of the significantly upregulated genes in XL (Table S1), indicating that genes that are upregulated in NT have a higher rank on average in XL than genes upregulated in XL do in NT. This is also consistent with biased performance between species. Genes with a low rank in XL tend to not be significantly upregulated in NT because genes that perform poorly in NT can only elevate the rank of genes that normally would have a lower rank if there were no bias. In contrast, intraspecific comparisons within XL using gDNA probemasks (Table S1) and the interspecific and intraspecific comparisons using the XB + XL perfect match probemask do not exhibit this pattern (Table S2).
